# Comparative Analysis of Zinc Finger Proteins Involved in Plant Disease Resistance

**DOI:** 10.1371/journal.pone.0042578

**Published:** 2012-08-15

**Authors:** Santosh Kumar Gupta, Amit Kumar Rai, Shamsher Singh Kanwar, Tilak R. Sharma

**Affiliations:** 1 National Research Centre on Plant Biotechnology, Indian Agricultural Research Institute, New Delhi, India; 2 Department of Biotechnology, Himachal Pradesh University, Summer-Hill, Shimla, India; Nanjing Agricultural University, China

## Abstract

A meta-analysis was performed to understand the role of zinc finger domains in proteins of resistance (*R*) genes cloned from different crops. We analyzed protein sequences of seventy *R* genes of various crops in which twenty six proteins were found to have zinc finger domains along with nucleotide binding sites - leucine rice repeats (NBS-LRR) domains. We identified thirty four zinc finger domains in the R proteins of nine crops and were grouped into 19 types of zinc fingers. The size of individual zinc finger domain within the *R* genes varied from 11 to 84 amino acids, whereas the size of proteins containing these domains varied from 263 to 1305 amino acids. The biophysical analysis revealed that molecular weight of *Pi54* zinc finger was lowest whereas the highest one was found in rice *Pib* zinc finger named as Transposes Transcription Factor (TTF). The instability (R^2^ = 0.95) and the aliphatic (R^2^ = 0.94) indices profile of zinc finger domains follows the polynomial distribution pattern. The pairwise identity analysis showed that the Lin11, Isl-1 & Mec-3 (LIM) zinc finger domain of rice blast resistance protein pi21 have 12.3% similarity with the nuclear transcription factor, X-box binding-like 1 (NFX) type zinc finger domain of Pi54 protein. For the first time, we reported that Pi54 (Pi-k^h^-Tetep), a rice blast resistance (R) protein have a small zinc finger domain of NFX type located on the C-terminal in between NBS and LRR domains of the R-protein. Compositional analysis depicted by the helical wheel diagram revealed the presence of a hydrophobic region within this domain which might help in exposing the LRR region for a possible *R-Avr* interaction. This domain is unique among all other cloned plant disease resistance genes and might play an important role in broad-spectrum nature of rice blast resistance gene *Pi54*.

## Introduction

The zinc finger proteins are a super family of proteins involved in numerous activities of plant growth and development and are also known to regulate resistance mechanism for various biotic and abiotic stresses [1], [2]. Any small, functional, freely folded domain in which coordination of one or more zinc ions required to stabilize its structure is known as zinc finger [3]. These domains are actively required to regulate various metabolic processes and stress conditions in plants [4], [5], [6]. The Zinc finger domains are widely dispersed in eukaryotic genomes [7], [4], [8], [9] and are actively involved in sequence specific binding to DNA/RNA and contribute in protein-protein recognitions [10]. The presence of zinc finger DNA binding domain in nucleotide binding sites-leucine rich repeats (NBS-LRR) class of proteins determines the regulatory function of this protein in stress conditions. These domains are basically transcription factor in origin which makes the protein as regulator. Zinc finger binds to DNA through the interaction of amino acids at the periphery of the zinc finger with base pairs at the center of the DNA double helix [11]. It is a compact protein domain, and its small size allows it to have close relation with DNA base pairs. Zinc fingers basically bind with nucleic acids for their function or participate in transcriptional or translational regulation processes [12]. These are classified into nine types according to their structural and functional variation. These are C2H2, C8, C6, C3HC4, C2HC, C2HC5, C4, C4HC3 and CCCH (C and H represent cysteine and histidine, respectively) [13], [14], [15], [16], [17]. The presence of zinc finger domain has been reported in many disease resistance genes cloned from various crops. These domains are LSD1 (C2C2), LOL1 (C2C2), Zat 12 (C2H2), Zat 7 (C2H2) & AtNFX1 (NF-X1) of Arabidopsis [18], StZFP1 (C2H2) of potato [8], and OsLSD1 (C2C2), OsLOL1 (C2C2), OsRING-1 (RING H2, RING HC), OsRFP1, OsDOS (CCCH), OsZFP (C2H2) & SRZ1(C2C2) of rice [19], [20]. These reports indicate that zinc finger motifs have important role in imparting host- plant resistance.

The NBS-LRRs are most prevalent class of R- proteins. These are basically three types such as TIR-NBS-LRR (TNL) and CC-NBS-LRR (CNL) and mixed type having either Toll/interleukin-1 receptor-like domain (TIR) or Coiled coil (CC ) domains or both in one protein [21], [22]. Proteins fused with these zinc finger domains make them as zinc finger proteins. Presence of these domains is required for the function of individual proteins under stress conditions. These domains are able to regulate the proteins like switches in rice and poplar [23], [24]. Sometimes, R proteins carry extensions at carboxy-terminus (C- terminal) with a typical WRKY DNA-binding domain and between linkers of NBS and LRR domains, respectively [25], [26].

Rice blast caused by *Magnaporthe oryzae* is one of the most important biotic stresses of rice resulting into huge yield loss every year [27]. The disease can be effectively managed by the resistance gene deployment. We have earlier identified and cloned a new rice blast resistance gene *Pi-k^h^* from *indica* rice cultivar, Tetep showing resistance to different strains of *M. oryzae* [28], [29]. The gene was only the third one to be cloned in the series of cloned rice blast resistance genes, after the cloning of *Pi-b* [30], and *Pi-ta* genes [31]. The gene was renamed as *Pi54*, after it was fine mapped at a slightly different location from the *Pi-k* cluster of the genes [32]. Functional validation of the gene has established that it confers a stable and high-level of resistance against geographically diverse strains of *M. oryzae* in India [33] and USA [34]. Expression analysis of the gene has revealed that it is induced by pathogen challenge. In turn, the gene was found to induce the synthesis of callose (β-1, 3 glucan) in response to pathogen challenge, suggesting its requirement in the initiation of a defense response cascade in the blast resistant plants [33]. Transcriptional and biochemical analysis revealed that rice transgenic lines containing *Pi54* single functional blast resistant gene show activation of a complex defense mechanism after pathogen inoculation [35]. The cloning of orthologue of *Pi54* gene has also been achieved from wild species of rice *O. rhizomatis* [36]. It has also been reported that *Pi54* protein contains an NBS-LRR domain in addition to a small zinc finger domain [37], [26].

The *R* genes are also categorized into separate categories on the basis of status and position of zinc finger domains. These domains are present either at N– terminal or C– terminal of the proteins encoded by *R* genes and along with NBS-LRR domain, play a crucial role in regulating expression of the genes involved in plant resistance [38], [39]. Many defense proteins of Arabidopsis and rice containing zinc finger domain have been shown to regulate programmed cell death (PCD). Despite having proven role in stress management in plants, the presence and involvement of zinc finger motifs along with NBS-LRR has not been analyzed in relation to plant disease resistance. The structural analysis of zinc finger domain present within these proteins is important for understanding the role of small protein domains that have diverse functions [13], [40].

The objectives of present study were (i) to analyze the presence of zinc finger domains in all the cloned plant disease resistance genes, (ii) to determine the probable structure of zinc finger domain of blast resistance protein Pi54 (iii) computational analysis of biophysical properties of zinc finger domains in the proteins of cloned *R* genes and (iv) comparative analysis of zinc finger proteins in relation to Pi54 protein.

## Results and Discussion

### Identification and amino acid composition analysis of zinc finger domains

**Figure 1:**
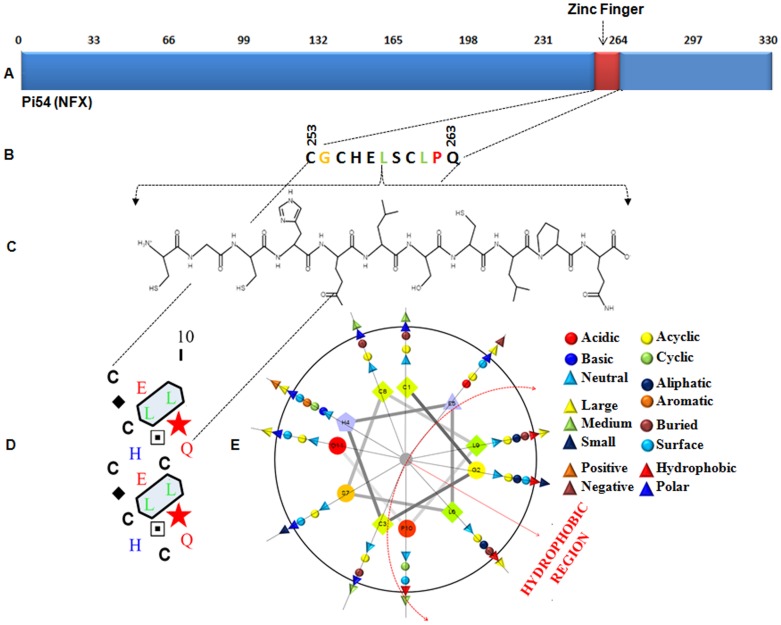
The structure of *Pi54* zinc finger domain. (A) Positional analysis of the domain showed that this domain is C- terminal in nature. The type of this zinc finger domain is NFX. (B) The amino acids, numbers and their positions in this domain. (C) Chemical structure of individual amino acids. (D) Secondary structure of zinc finger domain (E) Helical wheel diagram of *Pi54* zinc finger domain. The helical wheel is a plot of the amino acid residues around a potentially helical segment. The graphical representation showed the clustering of polar and/or non-polar residues toward one face of a helix.

The amino acid sequence of protein encoded by rice blast resistance gene *Pi54* was downloaded and analyzed using various bioinformatics tools along with a careful manual inspection. A small (11AA) zinc finger motif of nuclear transcription factor, X-box binding-like 1 (NFX) type was identified between the positions 253–263 amino acids in this protein. This domain is C– terminal in nature and integrated within LRR. Earlier *Pi-k^h^* (*Pi54*) gene responsible for the expression of this protein was found to be pathogen inducible in nature [35], [33], [26]. Amino acid composition analysis revealed that there are eight amino acids present within this domain with varying properties and mole percentage (Figure 1; Table 1). The amino acids in Pi54_ZnF domain were characterized on the basis of their side chains. These are negatively charged acidic (Glu E), non-polar aliphatic (G, L), polar uncharged (C, Q, S) and positively charged basic (H). The protein characteristics are believed to be related with the composition of amino acids and some of these structural factors were obtained due to the exchange of some amino acids [41].

**Table 1:** Amino acid compositional analysis of Pi54 Zinc finger domain.

S.No.	Amino Acid	Number	Mol%	R Group
1	Glu E	1	9.09	Negatively Charged , Acidic
2	Gly G	1	9.09	Non Polar Aliphatic
3	Leu L	2	18.18	Non Polar Aliphatic
4	Cys C	3	27.27	Polar Uncharged
5	Gln Q	1	9.09	Polar Uncharged
6	Pro P	1	9.09	Non-polar
7	Ser S	1	9.09	Polar Uncharged
8	His H	1	9.09	Positively Charged , Basic

#### Non-Polar amino acids

The nonpolar amino acids were characterized for having non polar atoms (only carbon and hydrogen) in their side chains. They include Glycine (Gly, G), Ala (Alanine, A), Val (Valine, V), Leu (Leucine, L), Ile (Isoleucine, I), Pro (Proline, P), and Met (Methionine, M). Presence of such residues makes domains more hydrophobic in nature. The hydrophobic amino acid residues can increase the rigidity and hydrophobicity of proteins [42]. Among the hydrophobic residues, Leu belongs to the aliphatic amino acids (Table 1). It has been found that aliphatic amino acids would contribute to the hydrophobic interaction and required to maintain the conformational stability in the inner part of the protein [43]. We identified two Leu residues in small zinc finger domain of Pi54 protein (Figure 1). More number of Leu residues result in higher average hydropathy and aliphatic index [44]. Besides, in Pi54 protein–Gly residues were also identified. Gly is known as the residue responsible to maintain or generate cavity in the inner part of protein structure [41]. These residues make domains more flexible for better folding in different way.

One Pro residue was also identified in the zinc finger domain of Pi54 protein. This residue contains a non-polar, uncharged R group (Figure 1). The Pro residue can only adopt a few configurations due to their pyrolidine ring and has the lowest conformational entropy. It thus restricts the configurations allowed for the preceding residue. Because of the presence of this residue rigidity and conformation in protein structure have been reported [45]. The Pro residue has been used to increase the protein stability in the several mutational studies and hence an increase in the stability of such domains of disease resistant proteins might be important for rice plant to resist more against *M. oryzae* [46].

#### Polar, uncharged amino acids

The polar nature of the side chain means that these amino acids are ready to interact with water (hydrophilic) and can form hydrogen bonds [42]. There are four numbers of polar uncharged amino acids (Cysteine C, Glutamine Q, Serine S and sometimes Histidine H with pK of 6.5) in small zinc finger domain of the Pi54 protein (Figure 1). These are the amino acids which possess oxygen, sulfur and/or nitrogen in the side chain and hence polar, but cannot have their side chain ionized and thus do not carry an overall charge. The amino acid Glutamine is known as thermolabile amino acids due to its tendency to undergo deamination at high temperature [41]. This indicates that presence of such residue makes the zinc finger domain of Pi54 more stable in various stress conditions; hence might help in maintaining the ability of rice plants to resist against the incoming *M. oryzae* pathogen under varied climatic conditions. The amino acid residue Serine is known as the best residues for interacting with the water molecules surrounding protein due to its hydrophilic nature [47]. The water molecules that are ready for interaction with these residues for hydrogen bond formation have been reported to release at high temperature. Hence, the protein structure around water-binding site changed to unstable which might increase the instability of proteins [48].

The side group of another polar, uncharged residue Cysteine present in zinc finger domain of Pi54 protein contains a sulfur atom (Figure 1). The sulfur group in Cysteine comes at the end of the hydrocarbon chain and therefore, has the potential to be more reactive. Cysteine is also known as thermolabile amino acids because it undergoes oxidation at high temperature like Methionine [49]. There are three Cysteine residues in Pi54 zinc finger domain. The proteins of maize, rice, and tomato contain 1.62%–1.69% cysteine whereas the yeast contained least cysteine (1.21%). More number of cysteine residues indicates existence of short-range intra-polypeptide chains interactions which plays important role in evolution [50]. Among the prokaryotes, Cyanobacteria, *E. coli*, *Psuedomonas aeruginosa*, and *Rhodobactor sphaeroides* contained 1.03%–1.13% cysteine. The cysteine contents of proteins of different species may be as low as 0.4%–0.5% in Archea. The extreme halophil *Haloarcula marismortui* and the thermophil *Thermus aquaticus* were reported to have the lowest 0.49% and 0.41%, respectively cysteine among the investigated species. The C-(X)_2_-C motifs present at position 253–260 of Pi54 protein are important domains of metal binding proteins [51].

#### Polar, charged amino acids

The negatively charged, acidic R group containing Glutamic acid and positively charged, basic amino acid, Histidine were also been identified in zinc finger domain of the Pi54 protein (Figure 1). The polar side chains of these residues can also carry a positive charge or negative charge and are therefore highly hydrophilic in nature. The charged amino acids would contribute to the electrostatic interaction, which is an important force for maintaining conformational stability in the outer part of the proteins [52], [53]. The conformational stability to expose the LRR domain for interaction with Avr proteins of the pathogen *M. oryzae* is necessary for *R* gene interactions [54]. Since the zinc finger domain of Pi54 protein is C– terminal in nature and integrated with LRR, it might be playing an important role in protein-protein interaction in rice-*M. oryzae* pathosystem. The charged amino acid residues are less labile and also retain the hydrogen bonding capacity. The charged residues may be involved in location of ion pairs within molecular structures which also appears to be important in determining protein stability [55]. The presence of these residues at the protein surface, increase ion interactions and enhances occurrence of salt bridges and ion pairs [56], [55]. These amino acids are important for the flexibility of the proteins [6].

The Helical wheel diagram gives a view of a helix from a protein sequence looking down the axis of the helix. It is useful for highlighting the amphipathicity and other properties of residues around a helix. The hydrophobic region of Pi54-ZnF domain which constitutes of two Leucine residues, one Proline and one Glycine has been represented by a helical wheel diagram (Figure 1). The helical wheel is a plot of the amino acid residues around a potentially helical segment. The method was developed to find helices with a hydrophobic face buried away from a polar solvent, with the graphical representation showing the clustering of polar and/or non- polar residues toward one face of a helix. Earlier, the helical wheel facilitated the identification of potential helical segments in protein sequences [57]. Besides, its applicability has also been expanded to designer proteins such as leucine zipper proteins [58] and studying trans-membrane proteins like G-protein- coupled receptors [59].

### Comparative analysis of proteins of *R* genes cloned from different crops

**Figure 2:**
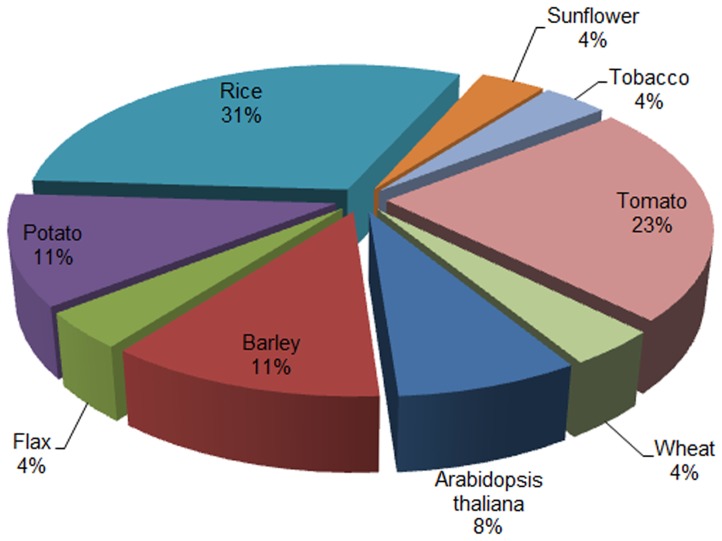
The distribution of zinc finger domains across different crops. The cloned *R* genes of various crops were found to have zinc finger domain in their proteins. The number of zinc finger domains represented by cloned *R* genes of each crop is shown in pie chart.

We analyzed protein sequences of 70 *R* genes cloned from different crops to delineate their zinc finger domain (Table S1 and Supplementary References, 1–69). The cloned disease resistance genes of the thirteen crops (Arabidopsis; Supplementary References (1–16), Barley (17–22), Pepper (23), Potato (24–27), Rice (28–43), Sunflower (44), Tomato (45–58), Tobacco (59), Wheat (60–63), Flax (64–66), Maize (67), Lettuce (68) and Beet (69)) were analyzed for identification and characterization of zinc finger domains in their respective proteins. The types of the proteins encoded by these *R* genes were varied in nature. The ‘SMART’ analysis revealed that twenty six genes contain zinc finger domains of different types, varying sizes and at different positions in their respective proteins. We found twenty six *R* genes from nine crops having nineteen types of zinc finger (Table 2). The *R* genes of rice represent 31% of all the zinc finger containing cloned *R* genes followed by tomato which has 23% proteins containing zinc finger domains. The *R* genes of wheat, sunflower, flax and tobacco were found to have zinc finger domains and constitute 4% of all the cloned *R* genes. Barley and potato represents 11% while Arabidopsis showed 8% share in this study of cloned *R* genes (Figure 2; Table S2). All zinc finger containing rice blast resistance genes encoded the NBS-LRR type of proteins except *Pi36* and *Pi5-1* which contains the CC-NBS-LRR type of proteins. The sunflower gene *pI8* was also found to contain zinc finger domain but is a CC-NBS-LRR type of protein. Tobacco *R* gene ‘*N*’ encoded the TIR-NBS-LRR type of protein also have the zinc finger domains. The six *R* genes of Tomato have zinc finger domain in their NBS-LRR, LZ-NBS-LRR and LRR types of the proteins (Table 2).

**Table 2:** Summary of various Zinc finger domains present in cloned *R* genes.

S No.	Crop name	Name of *R* gene	Protein length (aa)	Zinc finger type	Size of ZnF domain	Position of ZnF protein	Number of ZnF domains	Accession number	Disease resistance type	References
**1**	Arabidopsis	*SSI4*	1055	DBF	52	910–961	1	AAN86124.1	TIR-NBS-LRR	[92]
**2**	Arabidopsis	*RCY1*	361	RAD18	22	48–69	2	NP_001077963.1	RLKs	[93]
				PMZ	28	129–156				
**3**	Barley	*Mla1*	958	BED	49	386–434	1	AAG37354.1	CC-NBS-LRR	[94]
**4**	Barley	*Mla12*	961	BED	43	893–935	1	AAO43441.1	CC-NBS-LRR	[95]
**5**	Barley	*Rpg1*	837	TTF	84	145–228	2	AAM76922.1	RLKs	[96]
				C3H1	21	543–563				
**6**	Flax	*M*	1305	Rad18	16	290–305	1	AAB47618.1	NBS-LRR	[97]
**7**	Potato	*Rx2*	912	BED	47	346–392	1	CAB55838.1	NBS-LRR	[98]
**8**	Potato	*Gpa2Rx1*	937	BED	47	346–392	1	CAB50786.1	NBS-LRR	[99]
**9**	Potato	*Gro 1.4*	1136	CHCC	41	113–153	2	AAP44390.1	TIR-NBS-LRR	[100]
				CDGSH	31	688–718				
**10**	Rice	*Pib*	1251	U1	35	546–580	2	BAA76282.2	NBS-LRR	[30]
				TTF	83	60–142				
**11**	Rice	*Pi-ta*	928	UBP	43	537–579	1	AAK00132.1	NBS-LRR	[31]
**12**	Rice	*Pi36*	1056	CHCC	40	845–884	1	ABI64281.1	CC-NBS-LRR	[101]
**13**	Rice	*Pi54*	330	NFX	11	253–263	1	AAY33493.1	NBS-LRR	[26]
**14**	Rice	*Piz-t*	1033	PMZ	20	645–664	1	ABC73398.1	NBS-LRR	[102]
**15**	Rice	*Pi21*	263	LIM	39	11–49	1	BAG72124.1	NBS-LRR	[76]
**16**	Rice	*Pi5-1*	1025	TAZ	67	749–815	1	ACJ54697.1	CC-NBS-LRR	[78]
**17**	Rice	*Pi-2*	974	UBP	45	74–118	1	ABC94597	NBS-LRR	[102]
**18**	Sunflower	*pI8*	1279	CHCC	45	292–236	3	AAT08955.1	CC-NBS-LRR	[103]
				GATA	43	754–796				
				C2C2	31	1083–1113				
**19**	Tobacco	*N*	1128	C2H2	30	568–597	1	BAD12594	TIR-NBS-LRR	[104]
**20**	Tomato	*Mi-1*	1257	UBR1	56	1064–1119	1	AAC97933	NBS-LRR	[105]
**21**	Tomato	*Sw5-e*	1241	UBP	37	42–78	1	AAG31017	LZ-NBS-LRR	[106]
**22**	Tomato	*Cf-9*	863	PMZ	20	160–179	1	AAA65235.1	LRR	[107]
**23**	Tomato	*I2C*	373	U1	27	127–153	1	AAB63276	NBS-LRR	[15]
**24**	Tomato	*Hero*	1283	ZZ	44	171–214	2	CAD29729	NBS-LRR	[108]
**25**	Tomato	*Cf-4*	806	ZNF_C4	39	46–84	1	CAA05268.1	LRR	[109]
**26**	Wheat	*Lr10*	636	C2C2	38	32–69	2	AAC49629.1	RLKs	[110]
				ZZ	39	238–276				

### Analysis of zinc finger domain in R proteins

**Figure 3:**
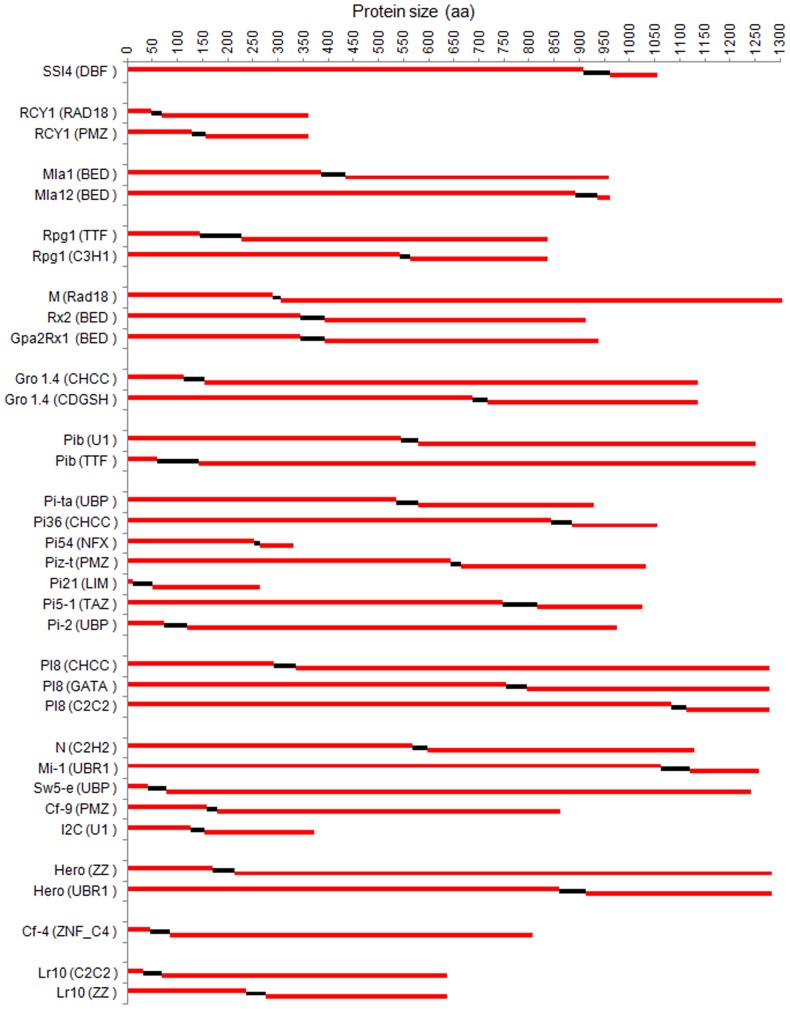
The architecture of zinc finger domains containing R proteins. The scale (0–1300) represents the size of proteins and their domains. The position and number of zinc finger domains in each *R* genes are also represented in the scale. The black broken lines indicate the zinc finger domains in individual R-proteins.

The zinc finger domains encoded by these proteins were used for further analysis. *R* genes of nine crops were further distributed into five families of eudicots and monocots representing their zinc finger domains (Table S2). Thirty four zinc finger sequences were found in twenty six *R* genes (Table S3). The domain architecture of these proteins depicts different types, size, position and number of zinc finger domains (Figure 3; Table 2). The blast resistance gene *Pi54* contain NFX type zinc finger domain in their protein. This domain is smallest and distinct from all others, though the smallest protein was encoded by *Pi21* of rice (Figure 3). The NFX1-type zinc finger proteins are a group of the human NFX1 transcription factors. This protein was identified as a protein that represses class II MHC (major histocompatibility complex) gene [60]. The NFX1-type zinc fingers containing proteins are found in protists, fungi, animals and plants. The NFX type zinc finger domain containing proteins are known to be involved in growth and survival of plants by managing reactive oxygen species (ROS), salicylic acid (SA), and also in biotic stress and abscisic acid (ABA) responses [61]. The only plant homologue AtNFXL1 of the NFX1 gene has been experimentally confirmed and found that it plays a crucial role in different stress responses [62], [63], [64]. In the present analysis, five genes were found to have two zinc finger domains whereas three zinc finger domains were obtained in one gene only. Twenty genes were found to contain only one zinc finger domains in their respective proteins (Table 2).

### Characteristic features of zinc finger domains in R-proteins

Zinc finger domain was deduced in a total of eight blast resistance genes of rice (Table 2). These genes were found to have ten different types of zinc finger domains in their respective proteins. The R- gene *Pib* encoded NBS-LRR protein along with two zinc finger domains U1 and TTF. Eight types of zinc fingers such as U1, TTF, UBP, CHCC, NFX, PMZ, LIM and TAZ were found in the rice R proteins (Table 2). The zinc finger type UBP was deduced in blast resistance genes *Pi-ta* and *Pi-2*. The size of zinc finger domains varies from 11 to 84 amino acid residues. The NFX, LIM and TAZ types were only found in the *R* genes cloned from rice. The U1-ZnF of *Pib* was reported in *I2C* gene of tomato whereas TTF was found in barley Rpg1 protein. The PMZ of rice deduced in Piz-t protein having length (20 AA) similar to PMZ of Tomato R protein Cf-9. This zinc finger domain is also present in RCY1 protein of barley. The LIM zinc finger deduced in Pi21 protein of rice is smallest protein amongst all the proteins of cloned genes in plants, whereas the zinc finger domain deduced in Pi54 protein was found to be the smallest amongst all plant proteins cloned till date. The *Pib* gene encoded protein of rice was found to have largest zinc finger domain TTF (83AA) among all the cloned *R* genes of rice though TTF of barley *R* gene *Rpg1* is largest (84 AA) among all the crops (Table 2, S3).

Among other crops, tomato contains six types of zinc finger domains like UBR1, UBP, PMZ, U1, ZZ and C4 in their disease resistance genes. The smallest zinc finger domain of tomato is PMZ (20AA) which was deduced in Cf-9 protein and is similar to the rice PMZ domain of blast resistance gene *Piz-t*. The UBR1 zinc finger of tomato protein Mi-1 is largest amongst all other cloned resistance proteins of tomato (Table 2, S3). Similarly, cloned R proteins of potato were found to have three types of zinc finger domains such as BED, CHCC and CDGSH. Of these, the zinc finger domain BED has also been deduced in *Mla1*& *Mla12* genes of barley.

### Biophysical characterization of zinc finger domains of R-proteins

The Protparam analysis revealed that these R-proteins have varying numbers and types of amino acids (Table S1, S3). This analysis also includes the molecular weight, theoretical PI, instability index, aliphatic index and hydropathicity index of all zinc finger domains present in *R* genes (Table S1, S4). The molecular weight of *Pi54* zinc finger NFX is 1189.3 Daltons. It was lowest among all, whereas the highest one was 9905 Daltons in *Pib* zinc finger TTF (Table S4). The isoelectric point (pI) of zinc fingers of cloned R- genes ranged from 4.0 (UBP of *Sw5-e*) to 10.66 (PMZ of *RCY1*). The isoelectric point (pI) is the pH at which the surface of protein is covered with charge but net charge of protein is zero. The pI of Pi54 zinc finger domain was found to be 5.24 (Table S4) which indicates that this domain is slightly acidic in nature. The calculated isoelectric point (pI) will be useful because at this pI, solubility is least and mobility in an electro focusing system is zero [65].

### The instability status of zinc finger proteins

**Figure 4:**
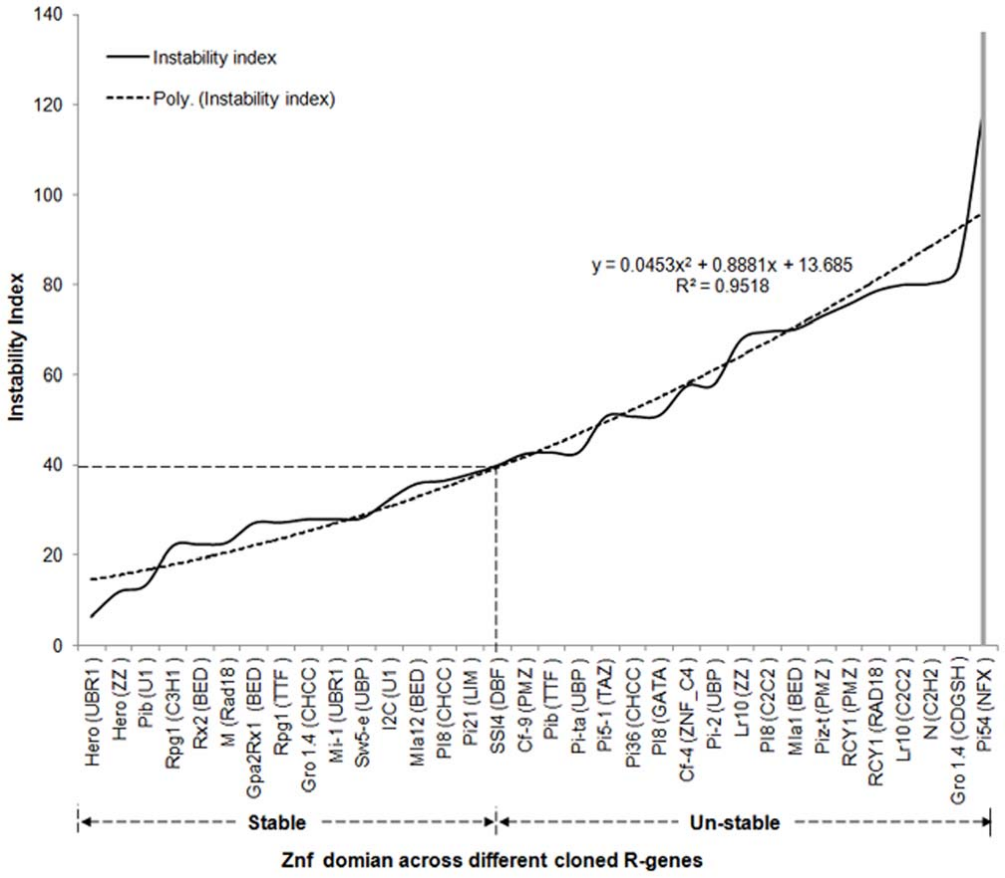
Instability index profile of identified zinc finger domain across cloned *R* gene. The data of 26 zinc finger *R* genes with their zinc finger types were included in this analysis.

The instability index value for the *R* gene zinc finger domains ranged from 6.37 to 120.89. The instability index of *Pi54* zinc finger domain was 120.89. This value indicates its highly unstable nature among all the cloned *R* genes. The zinc finger domain is arranged in their increasing order of stability profile which follows the polynomial trends (R^2^ = 0.95) (Figure 4; Table S4). The instability index affords an estimate of the stability of protein in a test tube. This method assigns a weight value of instability, which can be used to compute an instability index. A protein whose instability index smaller than 40 is predicted as stable where as a value above 40 predicts that the protein may be unstable [66].

### The aliphatic index of zinc finger domains of R proteins

**Figure 5:**
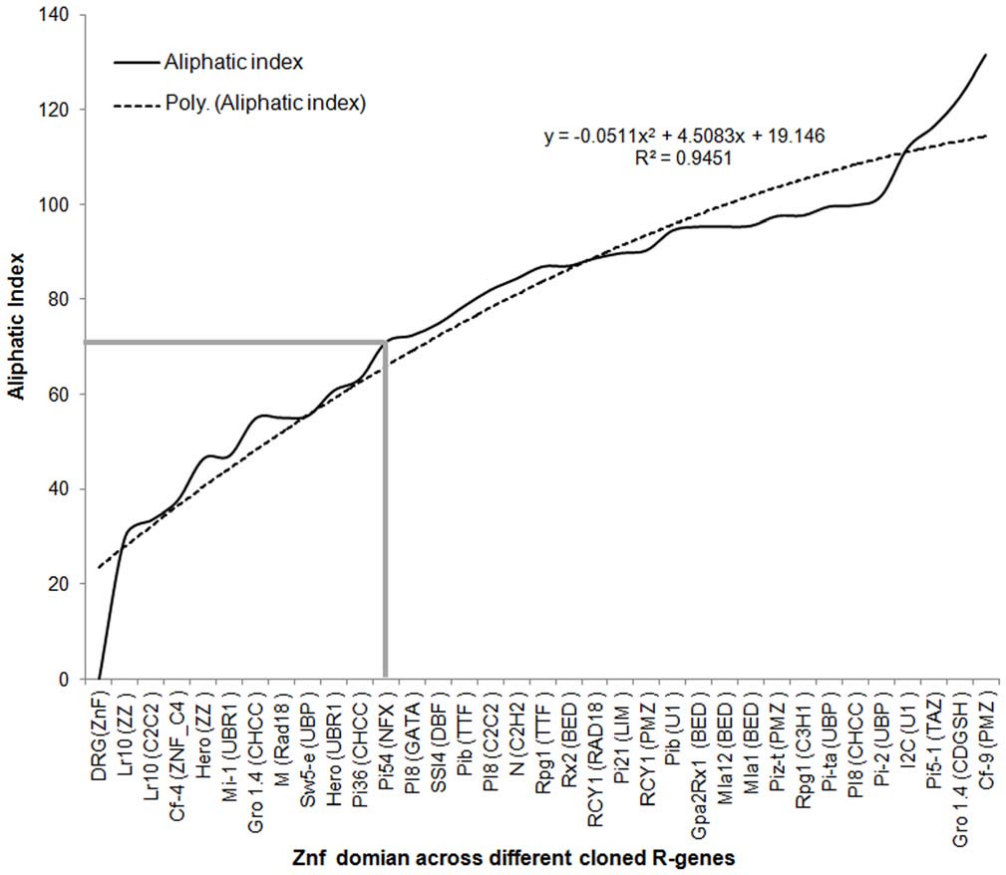
Aliphatic index profile of identified zinc finger domain across cloned *R* gene. The data of 26 zinc finger *R* genes with their zinc finger types were included in this analysis.

The aliphatic index is defined as the relative volume of a protein occupied by aliphatic side chains. The aliphatic index of the zinc finger sequences ranged from 30.0 to 122.58. The very high aliphatic index of zinc finger sequences indicates that these domains are stable at wide temperature range [67]. The aliphatic index of *Pi54* zinc finger domain ZnF_NFX was recorded as 70.91. This value indicates that the NFX domain of Pi54 is thermo-stable as well as flexible in nature. The high aliphatic index 122.58 was recorded for CDGSH zinc finger domain of *Gro 1-4* gene of potato. The lower thermal stability of ZZ zinc finger domain of *Lr10* gene of wheat indicates that this domain is very flexible than others. The aliphatic index profile follows the polynomial distribution pattern (R^2^ = 0.94) (Figure 5; Table S4). The aliphatic index (AI) occupied by aliphatic side chains (A, V, I and L) is regarded as a positive factor which is defined as the relative volume of a protein for the increase of thermal stability of globular proteins [68], [69]. The very high aliphatic index of zinc finger sequences indicates that these domains are stable at wide temperature range [67].

### Analysis of Grand Average of Hydropathy of zinc finger domains of R-proteins

**Figure 6:**
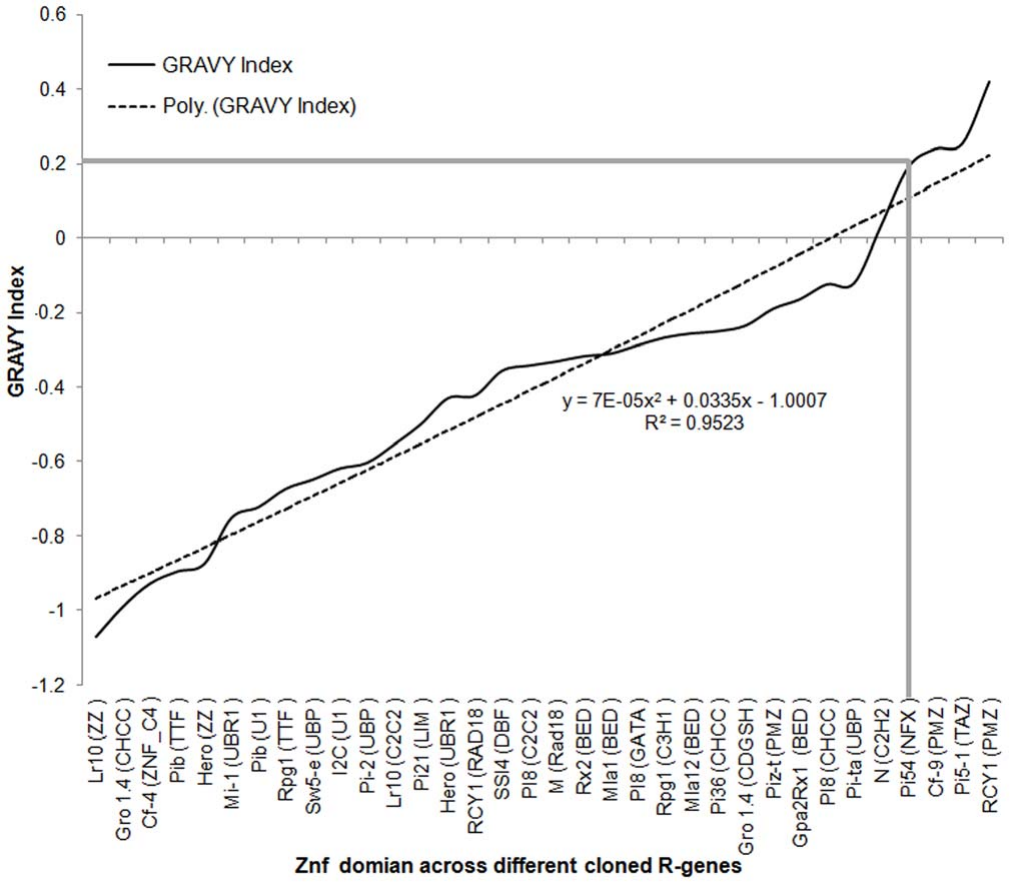
Grand Average of hydropathicity index profile of identified zinc finger domain across cloned *R* gene. The data of 26 zinc finger *R* genes with their zinc finger types were included in this analysis.

The Grand Average of Hydropathy (GRAVY) indices of zinc finger domain of cloned *R* genes ranged from −1.072 to 0.421 (Figure 6; Table S4). GRAVY values determined to provide a view of the hydrophobicity of the whole protein [70]. The GRAVY values usually vary in the range of ±2. Positive scores indicate hydrophobicity and negative scores indicate hydrophilicity. This low range of GRAVY value indicates the possibility of better interaction with water [67], [71]. The GRAVY value for *Pi54* ZnF domain was calculated as 0.191 (Figure 6) which indicates that this domain is hydrophobic in nature. This nature of the domain of R proteins makes them available for better interaction with their Avr counterparts as reported in case of Pto protein of tomato [72]. GRAVY values calculated for this domain of all the protein also follows the linear distribution pattern ((R^2^ = 0.95). The majority of zinc finger domains were hydrophilic in nature and some were found to hydrophobic.

### Phylogenetic relationship among zinc finger motifs

**Figure 7:**
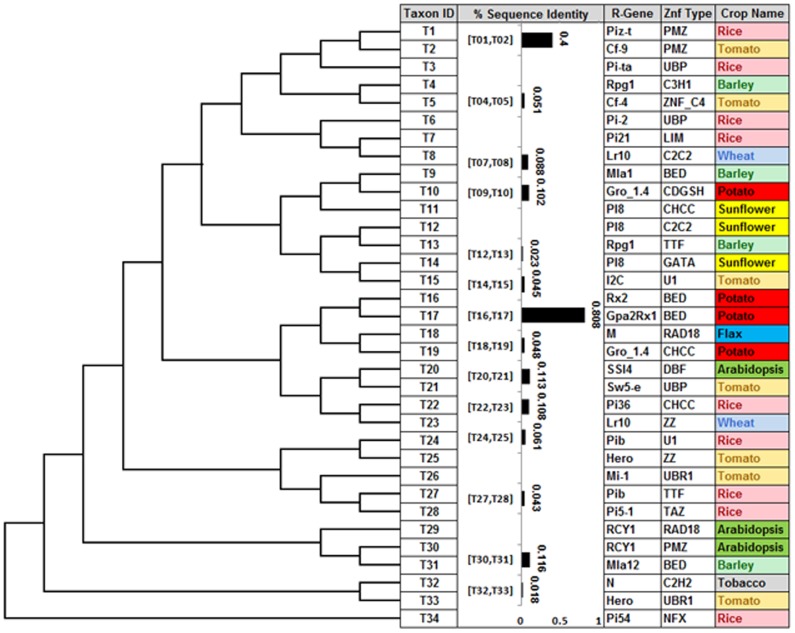
Maximum parsimony tree of 34 *R* genes containing zinc finger proteins. The bootstrap consensus tree (1000 replicates) is taken to represent the relationship between the taxa. Branches corresponding to partitions reproduced in less than 50% bootstrap replicates are collapsed. The percentage of replicate trees in which the associated taxa clustered together in the bootstrap test (1000 replicates) is shown next to the branches. The name of *R* gene, zinc finger types and crops are given on the termini of branches.

To establish a relationship between thirty four taxa of zinc finger domains, a maximum parsimony tree was constructed to identify the significant correlation among the different and highly divergent zinc fingers present in *R* genes. Fourteen possible groups of taxa having sequence similarities between each other were obtained (Figure 7). Further, percentage identity plot was calculated between each taxa which results a significant association with maximum parsimony inference between T1 & T2 (40%) and T16 & T17 (80.8%). The taxa T16 & T17 belong to potato *R* genes *Rx2* and *Gpa2/Rx1*. Both these genes contained BED zinc finger domains. The size of zinc finger domains in both the proteins is similar (47AA) but the size of both the NBS-LRR proteins is 912 and 937 AA, respectively (Table 2). This zinc finger domain was first reported in 2000 after two drosophila proteins named BEAF and DREF were identified [23]. These domains are probably involved in regulatory function of transcriptional control in plant disease resistance proteins [73]. Similarly the T1 & T2 taxa were represented by rice (*Piz-t*) and tomato (*Cf-9*). These are having sequence similarities between PMZ zinc fingers. The PMZ zinc finger size is 20 AA in both the proteins though the size of the protein in both the genes was 1033 AA (NBS-LRR) and 863 AA (LRR), respectively (Figure 7; Table 2). The PMZ (Plant Mutator Transposase) zinc finger is basically a transcription factor that is induced during the senescence and pathogen infection. These domains are present in AN1 like protein families. The PMZ domain containing proteins are induced by the abscisic acid and chitin stimuli [74], [75]. There are four groups of taxa having more than 10% parsimony inference between each other. These are T9 & T10 (10.2%), T20 & T21 (11.3%), T22 & T23 (10.8%) and T30 & T31 (11.6%). However, eight groups of taxa having less than 10% sequence similarities between each other were also obtained. The parsimony inference was not observed between zinc finger domain of *Pi54* and other genes. These findings indicate that the zinc finger domain of Pi54 protein is distinct amongst all the analyzed highly divergent zinc finger sequences of the proteins of plant disease resistance genes.

### Identity plot of zinc fingers of cloned R- genes in relation to *Pi54* gene

**Figure 8:**
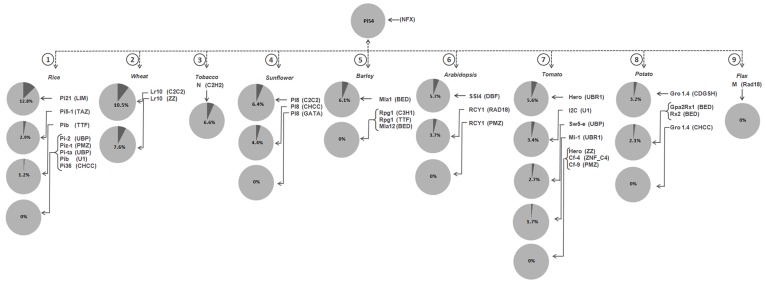
Identity plot of zinc finger domains. Identity plot of zinc fingers represents the identity analysis between amino acid sequences of Pi54 zinc finger domain and zinc finger domains present in cloned *R* genes of various crops.

This analysis encompasses the identity between the zinc finger domains of blast resistance gene *Pi54* and the genes cloned from other crops (Figure 8). The blast resistance genes cloned from rice were found to be closer to *Pi54*. The rice blast resistance protein pi21 is the smallest protein amongst all the zinc finger proteins which contains the LIM zinc finger domain [76]. This domain showed 12.3% sequence similarity (AA sequences) with zinc finger domain of Pi54 protein. The LIM domain is also a new functional motif deduced in *pi21* gene of rice and it contains a cysteine-rich motif of CX_2_ - CX_17–19_HX_2_CX_2_CX_2_CX_16–20_CX_2–3_C. It was reported that LIM- containing proteins have been implicated in the transcriptional regulation of cell-differentiation and growth regulation and serve as the site for protein-protein interaction [17]. The similarity in amino acid sequences of zinc finger domains between Pi54 and pi21 supports our hypothesis that the LRR integrated NFX zinc finger of Pi54 might be involved in protein-protein interaction. We obtained 2.9% similarity between TAZ zinc finger domain of Pi5-1 and NFX domain of Pi54 proteins. The transcriptional adapter zinc binding (TAZ) domains are important sites for protein-protein interactions [77]. The similarity obtained between TAZ zinc finger domains of *Pi5-1* also supports our hypothesis. It was also reported that both the genes *Pi5-1* [78] and *Pi54* [26], [33], [35] expressed constitutively at a basal level in both transgenic as well as susceptible native lines up to 48 hours post inoculation and induced by *M. oryzae* infection in later hours. The pathogen inducible nature of both the genes also supports this analysis that similar amino acids in these proteins might play some important role in *R-Avr* interactions. The TTF zinc finger domain of Pib protein showed only 1.2% similarity with Pi54. The UBP zinc finger of Pi-z, PMZ zinc finger domain of Piz-t, UBP zinc finger of Pita, U1 zinc finger of Pib and CHCC of Pi36 protein were found to have no similarity with Pi54 zinc finger domain. The Lr10 disease resistance protein of wheat was found to have two zinc finger domains namely C2C2 and ZZ and have 10.5% and 7.6% similarity with zinc finger domain of Pi54 protein, respectively. The C2C2 zinc finger domain is reported as novel zinc finger in many disease resistance proteins of various crops like *Arabidopsis* (*LSD1& LOL1*) and its homologs in rice *OsLSD1* and *OsLOL1* that negatively regulates programmed cell death (PCD) and plant defense signaling pathways [19], [30]. The similarity found between pairwise sequence alignment of zinc finger domains C2C2 and NFX showed that both are actively involved in negative regulation of up or down stream defense events. It has already been reported that NFX domains actively worked as negative regulators of various regulatory mechanisms which improves the physiological status of plants and supports growth and survival under various stress conditions. The expression of such type of zinc finger containing rice blast resistance gene *Pi54* was found under biotic stress conditions [64], [79], [35], [33], [26]. The ZZ zinc finger domain is a type of protein domain that was named because of its ability to bind two zinc ions [80]. The ZZ zinc finger domains containing proteins are found to be involved in protein-protein interactions and generation of hypersensitive responses [81]. The similarity between amino acid sequences of ZZ and NFX domains further supports the results of Pi54 zinc finger analysis. Tobacco *R* gene *N* having C2H2 zinc finger domain showed 6.6% similarity with NFX of *Pi54*. The C2H2 zinc finger proteins were mainly related to the plant development regulation or involved in various types of stress responses [82]. The one of the largest transcription factor families in eukaryotes are constituted by Cys2/His2-type zinc finger proteins [83]. Many stress-responsive C2H2-type zinc finger proteins have been identified in various plant species. Several studies have reported that C2H2-type zinc finger proteins are responsible for both the activation of some stress-related genes and enhanced tolerance to salt, dehydration, and/or cold stresses [82]. The C2H2 zinc finger containing proteins are also reported for their ROS scavenging nature and enhanced expression of defense response genes in plants [84]. Hence, C2H2 zinc finger of N protein having similarity with Pi54 zinc finger also supports our results.

The *R* gene of sunflower *pI8* was found to be having three zinc finger domains viz C2C2, CHCC and GATA. Two zinc finger domains C2C2 and CHCC showed 6.4 and 4.4% similarities with NFX domain of Pi54 protein, respectively. These domains in pI8 protein were also present at -C terminal like that of the zinc finger domain of Pi54 protein. The CHCC zinc finger domains of pI8 protein are the variants of the classical zinc fingers like C2C2 and C2H2 [85]. The formation of CHCC is due to replacement of one cysteine residue by histidine residue. These are short zinc-finger domains conserved from fungi to humans with a consensus sequence of Cx8Hx14Cx2C. Both the domains, NFX and CHCC are short in length, have three cysteine and one histidine residues and structural fold comprising a β-hairpin followed by a short α-helix that adopt two different conformations. This makes the structure of such type of zinc fingers highly divergent from other eukaryotic zinc fingers and these motifs are reported to have a group of DNA binding proteins from *Archea* [86]. However, the third zinc finger domain GATA of this gene showed no similarity with NFX-*Pi54*. There were no significant similarity between Pi54 and other zinc finger domains of barley proteins. The zinc finger domains of Tomato disease resistance proteins, viz., Hero, I2C, Sw5-e and Mi-1 have 5.6% and 3.4%, 2.7% and 1.7% similarity with Pi54 protein, respectively. The zinc finger domains of Arabidopsis and Potato R proteins showed 5.7 and 2.1% sequence similarities with NFX zinc finger domain of Pi54 protein, respectively. However, no similarity was found between Rad18 zinc finger domain of Flax M protein and NFX of Pi54 (Figure 8).

An identity matrix shows the proportion of identical residues between all of the sequences in the alignment as they are originally aligned. A total of 561 combinations of pairwise sequence identity were generated with the given data sets, out of which 90.55% of the combinations shows less than 10% sequence identity (which represents a significant statistical support of zinc finger sequence divergence) whereas 8.73% of the combinations shows an identity between 10–25% and the highest pairwise sequence identity (80.8%) exist between Gpa2Rx1_BED-Potato & Rx2_BED-Potato followed by Cf-9_PMZ-Tomato & Piz-t_PMZ-Rice (40.0%), Mla1_BED-Barley & Rx2_BED-Potato (38.7%) and Mla1_BED-Barley & Gpa2Rx1_BED-Potato (36.7%).

**Figure 9:**
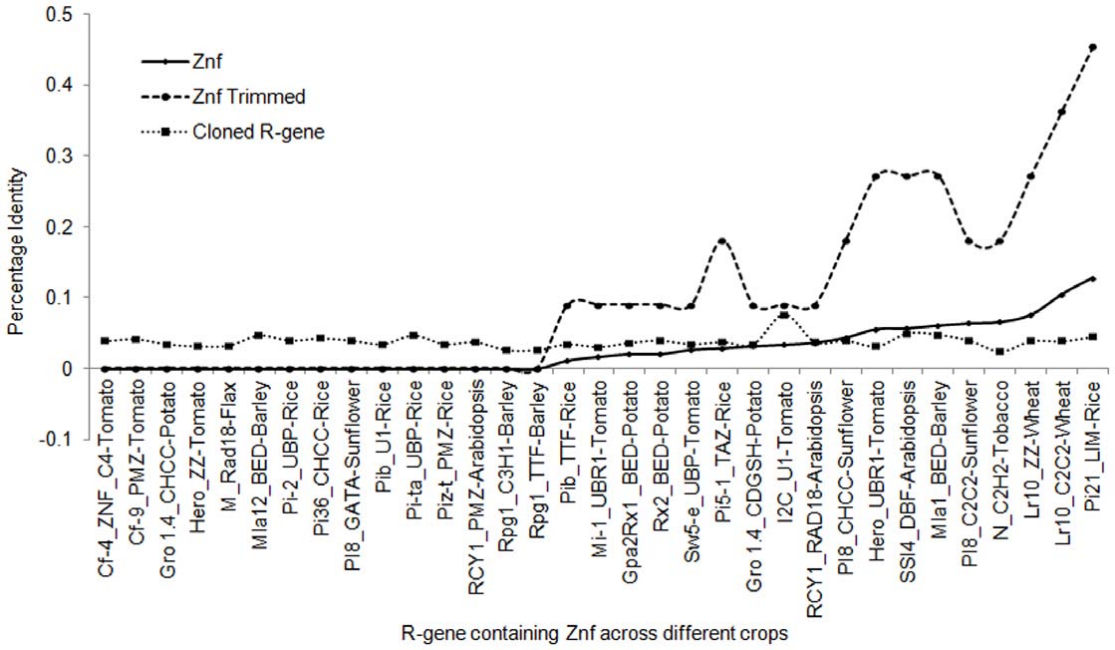
Pair-wise percentage identity profile. The Graphical representation showed the pair wise percentage identity profile of zinc finger domains of cloned *R* genes with respect to NFX domain of *Pi54* gene. This analysis includes the comparison between the complete protein sequences of zinc finger containing R-proteins, Zinc finger domains of these proteins and 11 amino acid trimmed sequences showed similarity with *Pi54* zinc finger domains. The X-axis of the graph represents the zinc finger containing cloned *R* genes across different crops whereas Y-axis represent the percentage identity profile between zinc fingers with respect to Pi54.

Pairwise identity matrix created from the zinc finger proteins, zinc finger domains and 11AA trimmed sequence aligned with Pi54_NFX-Rice is given in Figure 9. The analysis showed that Pi21 protein has 0.5% identity with Pi54. In case of zinc finger domain, LIM zinc finger of *pi21*gene of rice showed maximum identity (10%) with the zinc finger domain (NFX) of Pi54 protein (Figure 9). The 15 zinc finger domain sequences analyzed in the present study did not show any identity with zinc finger domain of Pi54 protein (Figure 9). A total of fifteen zinc finger domains of proteins (Pib, Mla-1, GPa2Rx1, Rx2, Sw5-e, Pi5-1, Gro1.4, I2C, RCY1, pI8 & N) were found with increasing identity with zinc finger domain of Pi54 blast resistance protein. Three zinc finger domains of wheat (Lr10_ZZ-wheat, Lr10_C2C2-wheat), and rice (pi21_LIM-rice) were found to be very close to NFX zinc finger domain of Pi54 protein. These domains are known to be actively involved in protein-protein interaction and as a potential regulator of various regulatory mechanisms, besides helping to maintain the physiological status of these proteins in various abiotic and biotic stresses [17], [64], [79]. After alignment of the eleven amino acids of the Pi54 zinc finger with other zinc finger domains as trimmed sequence, we found that LIM zinc finger domain of rice blast resistance pi21 protein showed maximum similarity (more than 40%) with zinc finger domain of rice blast resistance protein Pi54. The TTF zinc finger of Pib, UBR1 of Mla1, BED of the GPa2Rx1 and UBP of Sw5-e showed 10% identity to the Pi54 zinc finger while TAZ zinc finger of Pi5-1 showed 20% identity. The CHCC zinc finger of pI8, UBR1 of Hero, DBF of SSI4 and BED zinc finger of Mla1 showed 30% identity. The C4 zinc finger of Cf-4, and TTF of Rpg1 protein showed no (0%) identity with the Pi54 zinc finger (Figure 9).

## Conclusions

This study presents an analysis of plant disease resistance protein sequences in which zinc finger domains were found to be present along with other previously described domains like NBS and LRRs. The *Pi54* gene conferring durable resistance to blast disease in rice encodes a NBS-LRR protein along with a typical zinc finger domain. The zinc finger domain of Pi54 protein is NFX type and located on the C-terminal in between NBS and LRR domains of the R-protein. Compositional analysis depicted by the helical wheel diagram revealed the presence of a hydrophobic region within this domain which might help in exposing the LRR region for a possible *R-Avr* interaction. We also found the presence of different types of Zinc finger domains in rice blast resistance and in other plant disease resistance proteins. Maximum numbers of zinc finger domains were found in the proteins of disease resistance genes cloned from rice and tomato. Many disease resistance genes like *pI8* (Sunflower), *Pib* (Rice), *Lr10* (Wheat), *Gro1-4* (Potato), *RCY1* (Arabidopsis) and *Rpg1* (Barley) contains multiple number of zinc finger domains. The Pi54 protein contains the smallest zinc finger domain, despite the fact that smallest protein among these plant disease resistance proteins is pi21, another blast resistance gene. Instability, aliphatic and hydropathicity profile of these zinc finger domains gave a representation of the biochemical features of these proteins. We identified thirty four zinc finger domains in twenty six plant disease resistance proteins. These were found to be of nineteen different types of zinc fingers belonging to nine crops of five different families. Resistance proteins are known to play a crucial role in pathogen resistance by utilizing NBS and LRR domains for receiving signals from the pathogen. However, the presence of zinc finger domains, in combination with NBS-LRR domains in resistance proteins may reflect a major role of these domains in host- pathogen interaction.

## Materials and Methods

### *In- silico* analysis of zinc finger motif

The *in-silico* examination was performed to deduce the zinc finger domain in cloned *R* genes. The protein sequences of more than 70 genes present in National Centre for Biotechnology Information (NCBI) database (www.ncbi.nlm.nih.gov) were downloaded and analyzed (Table S1). The Expasy proteomic tool SMART (Simple Modular Architecture Research Tool) (www.smart.embl-heidelberg.de) was used for the identification of zinc finger motif [87], [88]. The identified zinc finger domain sequences were used for multiple sequence alignment using Clustal X version 1.83 (www.clustal.org) and Bio-edit 2.0 (http://www.mbio.ncsu.edu/bioedit) using default parameters [89], [90].

### Biophysical characterization

The structural and functional prediction of *Pi-k^h^* (*Pi54*) and other ZnF domains were studied using Expasy proteomic tool Protparam (http://us.expasy.org/tools/protparam.html) [70]. The physico-chemical parameters like Molecular weight, theoretical pI, instability index [66], aliphatic index [91] and grand average of hydropathicity (GRAVY) [70] were computed using Expasy's ProtParam Proteomics server. The Grand Average hydropathy (GRAVY) value for a peptide or protein is calculated as the sum of hydropathy values of all the amino acids, divided by the number of residues in the sequence.

### Phylogenetic analyses

The predicted zinc finger domains of R-proteins were further undertaken for phylogenetic analysis. The aligned sequences were inspected and adjusted manually to minimize the number of gaps and insertions. The manual adjustments were based on the sequence similarities. The phylogenetic tree was constructed according to the Neighbor-Joining method and visualized by MEGA program version 4.0 [56]. To validate the reproducibility of the branching pattern, bootstrap analysis (1000 replicates) and distance analysis were performed.

### Comparative pairwise identity profiling of *Pi54* zinc finger domain across different crops

For the pairwise identity between the zinc finger domain of blast resistance gene Pi54 and the genes cloned from other crops, 2-D pairwise identity data matrix was generated using Bio-Edit version 5.0.6 [89]. Further, 2-D pairwise identity data matrix was resolved with respect to Pi54 to delineate its comparative status in relation to other zinc finger domains across different crops.

## Supporting Information

Supplementary References List of References for Supplementary files.
(DOCX)

Table S1 The cloned plant disease resistance genes and their specific features.
(DOCX)

Table S2 Distribution of Zinc finger R-proteins across different crops.
(DOCX)

Table S3 Details of different Zinc finger domains across various crops cloned *R* genes.
(DOCX)

Table S4 Biophysical parameters of identified Zinc finger domains across various cloned *R* genes.
(DOCX)
