# Supplementary material for: Comparative Analysis of Zinc Finger Proteins Involved in Plant Disease Resistance
Source: PLoS One. 2012 Aug 15;7(8):e42578. doi: 10.1371/journal.pone.0042578 (PMC3419713; doi:10.1371/journal.pone.0042578)
Supplement: Supplementary References — List of References for Supplementary files. (DOCX) [file pone.0042578.s001.docx]

**Supplementary References**

1. Bent AF, Kunkel BN, Dahlbeck D, Brown KL, Schmidt R, et al. (1994) *RPS2* of *Arabidopsis thaliana*: A leucine-rich repeat class of plant disease resistance genes. Science 265: 1856-1860.
2. Gassmann W, Hinsch ME, Staskawicz BJ (1999) The Arabidopsis RPS4 bacterial-resistance gene is a member of the TIR-NBS-LRR family of disease-resistance genes. Plant J 20: 265-277.
3. Botella MA, Parker JE, Frost LN, Bittner-Eddy PD, Beynon JL, et al. (1998) Three genes of the Arabidopsis *RPP1* complex resistance locus recognize distinct *Peronospora parasitica* avirulence determinants. Plant Cell 10: 1847-1860.
4. van der Biezen EA, Freddie CT, Kahn K, Parker JE, Jones JDG (2002) Arabidopsis *RPP4* is a member of the *RPP5* multigene family of TIR-NB-LRR genes and confer downy mildew resistance through multiple signalling components. Plant J 29: 439-451.
5. Parker JE, Coleman MJ, Szabo V, Frost LN, Schmidt R, et al. (1997) The Arabidopsis downy mildew resistance gene *RPP5* shares similarity to the toll and interleukin-1 receptors with *N* and *L6*. The Plant Cell 9: 879-894.
6. Shirano Y, Kachroo P, Shah J, Klessig DF (2002) A gain-of-function mutation in an Arabidopsis Toll Interleukin1 receptor-nucleotide binding site-leucine-rich repeat type *R* gene triggers defense responses and results in enhanced disease resistance. Plant Cell 14: 3149-3162.
7. Deslandes L, Olivier J, Peeters N, Feng DX, Khounlotham M, et al. (2003) Physical interaction between RRS1-R, a protein conferring resistance to bacterial wilt, and PopP2, a type III effector targeted to the plant nucleus. Proc Natl Acad Sci U S A 100: 8024-8029.
8. Tör M, Brown D, Cooper A, Woods-Tör A, Sjölander K, et al. (2004) Arabidopsis downy mildew resistance gene *RPP27* encodes a receptor-like protein similar to CLAVATA2 and tomato *Cf-9*. Plant Physiol 135: 1100-1112.
9. Diener AC, Ausubel FM (2005) Resistance to *Fusarium oxysporum* 1, a dominant arabidopsis disease-resistance gene, is not race specific. Genetics 171: 305-321.
10. Xiao S, Ellwood S, Calis O, Patrick E, Li T, et al. (2001) Broad-spectrum mildew resistance in *Arabidopsis thaliana* mediated by *RPW8*. Science 291: 118-120.
11. Warren RF, Henk A, Mowery P, Holub E, Innes RW (1998) A mutation within the leucine-rich repeat domain of the Arabidopsis disease resistance gene *RPS5* partially suppresses multiple bacterial and downy mildew resistance genes. Plant Cell 10: 1439-1452.
12. Boyes DC, Nam J, Dangl JL (1998) The *Arabidopsis thaliana* *RPM1* disease resistance gene product is a peripheral plasma membrane protein that is degraded coincident with the hypersensitive response. Proc Natl Acad Sci U S A 95: 15849-15854.
13. McDowell JM, Dhandaydham M, Long TA, Aarts MGM, Goff S, et al. (1998) Intragenic recombination and diversifying selection contribute to the evolution of downy mildew resistance at the *RPP8* locus of Arabidopsis. The Plant Cell 10: 1861-1874.
14. Bittner-Eddy PD, Crute IR, Holub EB, Beynon JL (2000) *RPP13* is a simple locus in arabidopsis thaliana for alleles that specify downy mildew resistance to different avirulence determinants in *Peronospora parasitica*. Plant J 21: 177-188.
15. Takahashi H, Miller J, Nozaki Y, Takeda M, Shah J, et al. (2002) *RCY1*, an Arabidopsis thaliana *RPP8/HRT* family resistance gene, conferring resistance to cucumber mosaic virus requires salicylic acid, ethylene and a novel signal transduction mechanism. Plant J 32: 655-667.
16. Cooley MB, Pathirana S, Wu HJ, Kachroo P, Klessig DF (2000) Members of the arabidopsis *HRT/RPP8* family of resistance genes confer resistance to both viral and oomycete pathogens. Plant Cell 12: 663-676.
17. Büschges R, Hollricher K, Panstruga R, Simons G, Wolter M, et al. (1997) The barley *Mlo* gene: A novel control element of plant pathogen resistance. Cell 88: 695-705.
18. Zhou F, Kurth J, Wei F, Elliott C, Valè G, et al. (2001) Cell-Autonomous Expression of Barley *Mla1* Confers Race-Specific Resistance to the Powdery Mildew Fungus Via a *Rar1*-Independent Signaling Pathway. The Plant Cell 13: 337-350.
19. Halterman D, Zhou F, Wei F, Wise RP, Schulze-Lefert P (2001) The *MLA6* coiled-coil, NBS-LRR protein confers *AvrMla6*-dependent resistance specificity to *Blumeria graminis* f. sp. *hordei* in barley and wheat. Plant J 25: 335-348.
20. Shen Q, Zhou F, Bieri S, Haizel T, Shirasu K, et al. (2003) Recognition specificity and *RAR1/SGT1* dependence in barley *Mla* disease resistance genes to the powdery mildew fungus. Plant Cell 15: 732-744.
21. Halterman DA, Wise RP (2004) A single-amino acid substitution in the sixth leucine-rich repeat of barley *MLA6* and *MLA13* alleviates dependence on *RAR1* for disease resistance signaling. Plant J 38: 215-226.
22. Brueggeman R, Rostoks N, Kudrna D, Kilian A, Han F, et al. (2002) The barley stem rust-resistance gene *Rpg1* is a novel disease-resistance gene with homology to receptor kinases. Proc Natl Acad Sci U S A 99: 9328-9333.
23. Tai TH, Dahlbeck D, Clark ET, Gajiwala P, Pasion R, et al. (1999) Expression of the *Bs2* pepper gene confers resistance to bacterial spot disease in tomato. Proc Natl Acad Sci U S A 96: 14153-14158.
24. Bendahmane A, Querci M, Kanyuka K, Baulcombe DC (2000) Agrobacterium transient expression system as a tool for the isolation of disease resistance genes: Application to the *Rx2* locus in potato. Plant J 21: 73-81.
25. van der Vossen EA, van der Voort JN, Kanyuka K, Bendahmane A, Sandbrink H, et al. (2000) Homologues of a single resistance-gene cluster in potato confers resistance to distinct pathogens: A virus and a nematode. Plant J 23: 567-576.
26. Paal J, Henselewski H, Muth J, Meksem K, Menéndez CM, et al. (2004) Molecular cloning of the potato *Gro1-4* gene conferring resistance to pathotype Ro1 of the root cyst nematode *Globodera rostochiensis*, based on a candidate gene approach. Plant J 38: 285-297.
27. Ballvora A, Ercolano MR, Weiss J, Meksem K, Bormann CA, et al. (2002) The *R1* gene for potato resistance to late blight (*Phytophthora infestans*) belongs to the leucine zipper/NBS/LRR class of plant resistance genes. Plant J 30: 361-371.
28. Wang ZX, Yano M, Yamanouchi U, Iwamoto M, Monna L, et al. (1999) The *Pib* gene for rice blast resistance belongs to the nucleotide binding and leucine-rich repeat class of plant disease resistance genes. Plant J 19: 55-64.
29. Zhou B, Qu S, Liu G, Dolan M, Sakai H, et al. (2006) The eight amino-acid differences within three leucine-rich repeats between *Pi2* and *Piz-t* resistance proteins determine the resistance specificity to *Magnaporthe grisea*. Mol Plant Microbe Interact 19: 1216-1228.
30. Bryan GT, Wu K, Farrall L, Jia Y, Hershey HP, et al. (2000) A single amino acid difference distinguishes resistant and susceptible alleles of the rice blast resistance gene *Pi-ta*. Plant Cell 12: 2033-2046.
31. Liu X, Lin F, Wang L, Pan Q (2007) The *in-silico* map-based cloning of *Pi36*, a rice coiled-coil nucleotide-binding site leucine-rich repeat gene that confers race-specific resistance to the blast fungus. Genetics 176: 2541-2549.
32. Yoshimura S, Yamanouchi U, Katayose Y, Toki S, Wang Z, et al. (1998) Expression of *Xa1*, a bacterial blight-resistance gene in rice, is induced by bacterial inoculation. Proc Natl Acad Sci U S A 95: 1663-1668.
33. Song WY, Wang GL, Chen LL, Kim HS, Pi LY, et al. (1995) A receptor kinase-like protein encoded by the rice disease resistance gene, *Xa21*. Science 270: 1804-1806.
34. Lin F, Chen S, Que Z, Wang L, Liu X, et al. (2007) The blast resistance gene *Pi37* encodes a nucleotide binding site leucine-rich repeat protein and is a member of a resistance gene cluster on rice chromosome 1. Genetics 177: 1871-1880.
35. Sun X, Cao Y, Yang Z, Xu C, Li X, et al. (2004) *Xa26*, a gene conferring resistance to *Xanthomonas oryzae* pv. *oryzae* in rice encodes an LRR receptor kinase-like protein. Plant J 37: 517-527.
36. Qu S, Liu G, Zhou B, Bellizzi M, Zeng L, et al. (2006) The broad-spectrum blast resistance gene *Pi9* encodes a nucleotide-binding site-leucine-rich repeat protein and is a member of a multigene family in rice. Genetics 172: 1901-1914.
37. Gu K, Yang B, Tian D, Wu L, Wang D, et al. (2005) R gene expression induced by a type-III effector triggers disease resistance in rice. Nature 435: 1122-1125.
38. Chu Z, Yuan M, Yao J, Ge X, Yuan B, et al. (2006) Promoter mutations of an essential gene for pollen development result in disease resistance in rice. Genes Dev 20: 1250-1255.
39. Ashikawa I, Hayashi N, Yamane H, Kanamori H, Wu J, et al. (2008) Two adjacent nucleotide-binding site-leucine-rich repeat class genes are required to confer *Pikm*-specific rice blast resistance. Genetics 180: 2267-2276.
40. Shang J, Tao Y, Chen X, Zou Y, Lei C, et al. (2009) Identification of a new rice blast resistance gene, *Pid3*, by genome wide comparison of paired nucleotide-binding site--leucine-rich repeat genes and their pseudogene alleles between the two sequenced rice genomes. Genetics 182: 1303-1311.
41. Lee S, Song M, Seo Y, Kim H, Ko S, et al. (2009) Rice *Pi5*-mediated resistance to *Magnaporthe oryzae* requires the presence of two coiled-coil-nucleotide-binding-leucine-rich repeat genes. Genetics 181: 1627-1638.
42. Sharma TR, Madhav MS, Singh BK, Shanker P, Jana TK, et al. (2005) High-resolution mapping, cloning and molecular characterization of the *Pi-k^h^* gene of rice, which confers resistance to *Magnaporthe grisea*. Mol Genet Genomics 274: 569-578.
43. Fukuoka S, Saka N, Koga H, Ono K, Shimizu T, et al. (2009) Loss of function of a proline-containing protein confers durable disease resistance in rice. Science 325: 998-1001.
44. Radwan O, Mouzeyar S, Nicolas P, Bouzidi MF (2005) Induction of a sunflower CC-NBS-LRR resistance gene analogue during incompatible interaction with *Plasmopara halstedii*. J Exp Bot 56: 567-575.
45. Salmeron JM, Oldroyd GE, Rommens CM, Scofield SR, Kim HS, et al. (1996) Tomato *Prf* is a member of the leucine-rich repeat class of plant disease resistance genes and lies embedded within the *Pto* kinase gene cluster. Cell 86: 123-133.
46. Ori N, Eshed Y, Paran I, Presting G, Aviv D, et al. (1997) The *I2C* family from the wilt disease resistance locus I2 belongs to the nucleotide binding, leucine-rich repeat superfamily of plant resistance genes. Plant Cell 9: 521-532.
47. Milligan SB, Bodeau J, Yaghoobi J, Kaloshian I, Zabel P, et al. (1998) The root knot nematode resistance gene *Mi* from tomato is a member of the leucine zipper, nucleotide binding, leucine-rich repeat family of plant genes. Plant Cell 10: 1307-1320.
48. Jablonska B, Ammiraju JSS, Bhattarai KK, Mantelin S, de Ilarduya OM, et al. (2007) The *Mi-9* gene from *Solanum arcanum* conferring heat-stable resistance to root-knot nematodes is a homolog of *Mi-1*. Plant Physiol 143: 1044-1054.
49. Brommonschenkel SH, Frary A, Frary A, Tanksley SD (2000) The broad-spectrum tospovirus resistance gene *Sw5-e* of tomato is a homolog of the root-knot nematode resistance gene *Mi*. Mol Plant-Microbe Interact 13: 1130-1138.
50. Jones DA, Thomas CM, Hammond-Kosack KE, Balint-Kurti PJ, Jones JD (1994) Isolation of the tomato *Cf-9* gene for resistance to *Cladosporium fulvum* by transposon tagging. Science 266: 789-793.
51. Ernst K, Kumar A, Kriseleit D, Kloos D, Phillips MS, et al. (2002) The broad-spectrum potato cyst nematode resistance gene (*hero*) from tomato is the only member of a large gene family of NBS-LRR genes with an unusual amino acid repeat in the LRR region. Plant J 31: 127-136.
52. Parniske M, Hammond-Kosack KE, Golstein C, Thomas CM, Jones DA, et al. (1997) Novel disease resistance specificities result from sequence exchange between tandemly repeated genes at the *Cf-4/9* locus of tomato. Cell 91: 821-832.
53. Westerink N, Brandwagt BF, de Wit, Pierre JGM, Joosten MHAJ (2004) *Cladosporium fulvum* circumvents the second functional resistance gene homologue at the *Cf-4* locus (*Hcr9-4E*) by secretion of a stable *avr4E* isoform. Mol Microbiol 54: 533-545.
54. Dixon MS, Jones DA, Keddie JS, Thomas CM, Harrison K, et al. (1996) The tomato *Cf-2* disease resistance locus comprises two functional genes encoding leucine-rich repeat proteins. Cell 84: 451-459.
55. Dixon MS, Hatzixanthis K, Jones DA, Harrison K, Jones JD (1998) The tomato *Cf-5* disease resistance gene and six homologs show pronounced allelic variation in leucine-rich repeat copy number. The Plant Cell 10: 1915-1925.
56. Kawchuk LM, Hachey J, Lynch DR, Kulcsar F, van Rooijen G, et al. (2001) Tomato *Ve* disease resistance genes encode cell surface-like receptors. Proc Natl Acad Sci U S A 98: 6511-6515.
57. Schornack S, Ballvora A, Gürlebeck D, Peart J, Baulcombe D, et al. (2004) The tomato resistance protein Bs4 is a predicted non-nuclear TIR-NB-LRR protein that mediates defense responses to severely truncated derivatives of *AvrBs4* and overexpressed *AvrBs3*. Plant J 37: 46-60.
58. Martin GB, Frary A, Wu T, Brommonschenkel S, Chunwongse J, et al. (1994) A member of the tomato *Pto* gene family confers sensitivity to fenthion resulting in rapid cell death. Plant Cell 6: 1543-1552.
59. Whitham S, Dinesh-Kumar SP, Choi D, Hehl R, Corr C, et al. (1994) The product of the tobacco mosaic virus resistance gene *N*: similarity to toll and the interleukin-1 receptor. Cell 78: 1101-1115.
60. Yahiaoui N, Srichumpa P, Dudler R, Keller B (2004) Genome analysis at different ploidy levels allows cloning of the powdery mildew resistance gene *Pm3b* from hexaploid wheat. Plant J 37: 528-538.
61. Feuillet C, Schachermayr G, Keller B (1997) Molecular cloning of a new receptor-like kinase gene encoded at the *Lr10* disease resistance locus of wheat. Plant J 11: 45-52.
62. Lagudah ES, Moullet O, Appels R (1997) Map-based cloning of a gene sequence encoding a nucleotide-binding domain and a leucine-rich region at the *Cre3* nematode resistance locus of wheat. Genome 40: 659-665.
63. De Majnik J, Ogbonnaya FC, Moullet O, Lagudah ES (2003) The *Cre1* and *Cre3* nematode resistance genes are located at homeologous loci in the wheat genome. Mol Plant Microbe Interact 16: 1129-1134.
64. Lawrence GJ, Finnegan EJ, Ayliffe MA, Ellis JG (1995) The *L6* gene for flax rust resistance is related to the Arabidopsis bacterial resistance gene *RPS2* and the tobacco viral resistance gene *N*. Plant Cell 7: 1195-1206.
65. Ellis JG, Lawrence GJ, Luck JE, Dodds PN (1999) Identification of regions in alleles of the flax rust resistance gene *L* that determine differences in gene-for- gene specificity. The Plant Cell 11: 495-506.
66. Anderson PA, Lawrence GJ, Morrish BC, Ayliffe MA, Finnegan EJ, et al. (1997) Inactivation of the flax rust resistance gene *M* associated with loss of a repeated unit within the leucine-rich repeat coding region. The Plant Cell 9: 641-651.
67. Collins N, Drake J, Ayliffe M, Sun Q, Ellis J, et al. (1999) Molecular characterization of the maize *Rp1-D* rust resistance haplotype and its mutants. Plant Cell 11: 1365-1376.
68. Meyers BC, Chin DB, Shen KA, Sivaramakrishnan S, Lavelle DO, et al. (1998) The major resistance gene cluster in lettuce is highly duplicated and spans several megabases. Plant Cell 10: 1817-1832.
69. Cai D, Kleine M, Kifle S, Harloff HJ, Sandal NN, et al. (1997) Positional cloning of a gene for nematode resistance in sugar beet. Science 275: 832-834.
