# Supplementary material for: Comparative Analysis of Zinc Finger Proteins Involved in Plant Disease Resistance
Source: PLoS One. 2012 Aug 15;7(8):e42578. doi: 10.1371/journal.pone.0042578 (PMC3419713; doi:10.1371/journal.pone.0042578)
Supplement: Table S1 — The cloned plant disease resistance genes and their specific features. (DOCX) [file pone.0042578.s002.docx]

| **Table S1** The cloned plant disease resistance genes and their specific features | | | | | | |
| --- | --- | --- | --- | --- | --- | --- |
|  |  |  |  |  |  |  |
| **S No** | **Gene Name** | **Host** | **Pathogen** | **Protein type** | **Accession No** | **References** |
| 1 | *RPS2* | Arabidopsis | *Pseudomonas syringae* | CC-NBS-LRR | NP_194339.1 | [1] |
| 2 | *RPS4* | Arabidopsis | *Pseudomonas syringae* | TIR-NBS-LRR | NP_199338.1 | [2] |
| 3 | *RPP1* | Arabidopsis | *Peronospora parasitica* | TIR-NBS-LRR | AF98962.1 | [3] |
| 4 | *RPP4* | Arabidopsis | *Peronospora parasitica* | TIR-NBS-LRR | NP_193420.2 | [4] |
| 5 | *RPP5* | Arabidopsis | *Peronospora parasitica* | TIR-NBS-LRR | NP_193428.2 | [5] |
| 6 | *SSI4* | Arabidopsis | *Pseudomonas syringae* | TIR-NBS-LRR | AAN86124.1 | [6] |
| 7 | *RRS1-R* | Arabidopsis | *Ralstonia solanacearum* | WRKY-TIR-NBS-LRR | NP_199339.2 | [7] |
| 8 | *RPP27* | Arabidopsis | *Peronospora parasitica* | Receptor like protein | NP_175849.2 | [8] |
| 9 | *RFO1* | Arabidopsis | *Fusarium oxysporum* | Receptor like kinase | NP_178085.1 | [9] |
| 10 | *Rpw8* | Arabidopsis | *Erysiphe cruciferum* | TM-CC | AAK09266.1 | [10] |
| 11 | *RPS5* | Arabidopsis | *Pseudomonas syringae* | LZ-NBS-LRR | NP_172686.1 | [11] |
| 12 | *RPM1* | Arabidopsis | *Pseudomonas syringae* | CC-NBS-LRR | NP_187360.1 | [12] |
| 13 | *RPP8/HRT* | Arabidopsis | *Peronospora parasitica* | CC-NBS-LRR | NP_199160.1 | [13] |
| 14 | *RPP13* | Arabidopsis | *Peronospora parasitica* | CC-NBS-LRR | NP_190237.1 | [14] |
| 15 | *RCY1* | Arabidopsis | *Cucumber mosaic virus* | CC-NBS-LRR | NP_001077963.1 | [15] |
| 16 | *RPP/HRT* | Arabidopsis | *Turnip crinkle virus* | CC-NBS-LRR | AAF36987.1 | [16] |
| 17 | *Mlo* | Barley | *Erysiphe graminis* | TM | AAM14414.1 | [17] |
| 18 | *Mla1* | Barley | *Blumeria graminis* | CC-NBS-LRR | AAG37354.1 | [18] |
| 19 | *Mla6* | Barley | *Blumeria graminis* | CC-NBS-LRR | ABI13384.1 | [19] |
| 20 | *Mla12* | Barley | *Blumeria graminis* | CC-NBS-LRR | AAO43441.1 | [20] |
| 21 | *Mla13* | Barley | *Blumeria graminis* | CC-NBS-LRR | AAO16000.1 | [21] |
| 22 | *Rpg1* | Barley | *Puccinia graminis* | Protein kinase | AF509747 | [22] |
| 23 | *Bs2* | Pepper | *Xanthomonas campestris* | CC-NBS-LRR | AF202179 | [23] |
| 24 | *Rx2* | Potato | *Potato virus X* | CC-NBS-LRR | CAB55838.1 | [24] |
| 25 | *Gpa2/Rx1* | Potato | *Globodera pallida* | CC-NBS-LRR | CAB50786.1 | [25] |
| 26 | *Gro 1-4* | Potato | *Globodera rostochinensis* | TIR-NBS-LRR | AY196151 | [26] |
| 27 | *R1* | Potato | *Phytophthora infestans* | CC-NBS-LRR | AF447489.1 | [27] |
| 28 | *Pib* | Rice | *Magnaporthe oryzae* | CC-NBS-LRR | BAA76282.2 | [28] |
| 29 | *Pi-2/Piz-t* | Rice | *Magnaporthe oryzae* | NBS-LRR | ABC94597, ABC73398.1 | [29] |
| 30 | *Pi-ta* | Rice | *Magnaporthe oryzae* | CC-NBS-LRR | AAK00132.1 | [30] |
| 31 | *Pi36* | Rice | *Magnaporthe oryzae* | CC-NBS-LRR | ABI64281.1 | [31] |
| 32 | *Xa1* | Rice | *Xanthomonas oryzae* | CC-NBS-LRR | BAA25068.1 | [32] |
| 33 | *Xa21* | Rice | *Xanthomonas oryzae* | LRR-Ser/Thr protein kinase | BAE93933.1 | [33] |
| 34 | *Pi37* | Rice | *Magnaporthe oryzae* | NBS-LRR | ABI94578.1 | [34] |
| 35 | *Xa26* | Rice | *Xanthomonas oryzae* | LRR receptor-like kinase | ABD84048.1 | [35] |
| 36 | *Pi9* | Rice | *Magnaporthe oryzae* | NBS-LRR | ABB88855.1 | [36] |
| 37 | *Xa27* | Rice | *Xanthomonas oryzae* | NO homolog | AAY54165.1 | [37] |
| 38 | *Xa13* | Rice | *Xanthomonas oryzae* | Homolog of nodulin MtN3 | ABD78942.1 | [38] |
| 39 | *Pikm* | Rice | *Magnaporthe oryzae* | NBS-LRR | BAG72135.1 | [39] |
| 40 | *Pid3* | Rice | *Magnaporthe oryzae* | NBS-LRR | FJ745368.1 | [40] |
| 41 | *Pi5* | Rice | *Magnaporthe oryzae* | NBS-LRR | ACJ54697.1 | [41] |
| 42 | *Pi-k^h^*(*Pi54*) | Rice | *Magnaporthe oryzae* | NBS-LRR | AAY33493.1 | [42] |
| 43 | *Pi21* | Rice | *Magnaporthe oryzae* | NBS-LRR | BAG72124.1 | [43] |
| 44 | *pl8* | Sunflower | *Plasmopara halstedii* | CC-NBS-LRR | AY490793 | [44] |
| 45 | *Prf* | Tomato | *Pseudomonas syringae* | CC-NBS-LRR | AAV80420 | [45] |
| 46 | *I2* | Tomato | *Fusarium oxysporum* | CC-NBS-LRR | ABB00396 | [46] |
| 47 | *Mi-1* | Tomato | *Meloidogyne javanica* | CC-NBS-LRR | AAC97933 | [47] |
| 48 | *Mi-9* | Tomato | *Meloidogyne javanica* | CC-NBS-LRR | U159336.1 | [48] |
| 49 | *Sw5-e* | Tomato | *Tospovirus* | CC-NBS-LRR | AAG31017 | [49] |
| 50 | *Cf-9* | Tomato | *Cladosporium fulvum* | LRR-TM | AAA65235.1 | [50] |
| 51 | *Cf-2* | Tomato | *Cladosporium fulvum* | LRR-TM | 2207203A | [51] |
| 52 | *Cf-4* | Tomato | *Cladosporium fulvum* | LRR-TM | CAA05271.1 | [52] |
| 53 | *Hcr9-4E* | Tomato | *Cladosporium fulvum* | LRR-TM | AAT28196.1 | [53] |
| 54 | *Cf-2/5* | Tomato | *Cladosporium fulvum* | LRR-TM | AF053993 | [54, 55] |
| 55 | *Ve* | Tomato | *Verticillium wilt* | LRR-TM | AF272367.1 | [56] |
| 56 | *Bs4* | Tomato | *Xanthomonas campestris* | TIR-NBS-LRR | AAR21295 | [57] |
| 57 | *I2C* | Tomato | *Fusarium oxysporum* | LRR-TM | AAB63276 | [46] |
| 58 | *Hero* | Tomato | *Globodera rostochinensis* | NBS-LRR | CAD29729 | [51] |
| 59 | *Pto* | Tomato | *Pseudomonas syringae* | Ser/Thr Protein kinase | AAB47421 | [58] |
| 60 | *N* | Tobacco | *Tobacco mosaic virus* | TIR-NBS-LRR | BAD12594 | [59] |
| 61 | *Pm3b* | Wheat | *Blumeria graminis* | CC-NBS-LRR | AAQ96158.1 | [60] |
| 62 | *Lr10* | Wheat | *Puccinia triticina* | CC-NBS-LRR | AAC49629.1 | [61] |
| 63 | *Cre3* | Wheat | *Heterodera avenae* | NBS-LRR | AAC05834.2 | [62] |
| 64 | *Cre1* | Wheat | *Heterodera avenae* | NBS-LRR | AY124651 | [63] |
| 65 | *L6* | Flax | *Melampsora lini* | TIR-NBS-LRR | U27081 | [64] |
| 66 | *L,L1-L11,LH* | Flax | *Melampsora lini* | TIR-NBS-LRR | AF093638 AF093639 AF093641 AF093649 | [65] |
| 67 | *M* | Flax | *Melampsora lini* | TIR-NBS-LRR | U73916 | [66] |
| 68 | *Rp1* | Maize | *Puccinia sorghi* | CC-NBS-LRR | AF107293 | [67] |
| 69 | *Dm3* | Lettuce | *Bremia lactucae* | CC-NBS-LRR | AY206016.1 | [68] |
| 70 | *Hsl^pro^* | Beet | *Heterodera schachtii* | LRR-TM | U79733 | [69] |
