# Supplementary material for: Comparative Analysis of Zinc Finger Proteins Involved in Plant Disease Resistance
Source: PLoS One. 2012 Aug 15;7(8):e42578. doi: 10.1371/journal.pone.0042578 (PMC3419713; doi:10.1371/journal.pone.0042578)
Supplement: Table S2 — Distribution of Zinc finger R-proteins across different crops. (DOCX) [file pone.0042578.s003.docx]

| **Table S2** Distribution of Zinc finger R-proteins across different crops | | | | | |
| --- | --- | --- | --- | --- | --- |
|  | | | | | |
| **S No** | **Crop name** | **Class** | **Family** | **No. of R-protein sequence containing Znf** | **Count of different Znf** |
| 1 | *Arabidopsis thaliana* | Eudicots | *Brassicaceae* | 2 | 3 |
| 2 | Barley | Monocots | *Poaceae* | 3 | 3 |
| 3 | Flax | Eudicots | *Linaceae* | 1 | 1 |
| 4 | Potato | Eudicots | *Solanaceae* | 3 | 3 |
| 5 | Rice | Monocots | *Poaceae* | 8 | 8 |
| 6 | Sunflower | Eudicots | *Asteraceae* | 1 | 3 |
| 7 | Tobacco | Eudicots | *Solanaceae* | 1 | 1 |
| 8 | Tomato | Eudicots | *Solanaceae* | 6 | 6 |
| 9 | Wheat | Monocots | *Poaceae* | 1 | 2 |
