# Supplementary material for: Comparative Analysis of Zinc Finger Proteins Involved in Plant Disease Resistance
Source: PLoS One. 2012 Aug 15;7(8):e42578. doi: 10.1371/journal.pone.0042578 (PMC3419713; doi:10.1371/journal.pone.0042578)
Supplement: Table S3 — Details of different Zinc finger domains across various crops cloned R genes. (DOCX) [file pone.0042578.s004.docx]

| **Table S3** Details of different Zinc finger domains across various crops cloned R-genes | | | | |
| --- | --- | --- | --- | --- |
|  |  |  |  |  |
| **S No** | **Crop name** | **Name of the *R* gene** | **Zinc Finger type** | **Sequences (N-C) of Zn Finger domain** |
| 1 | Arabidopsis | *SSI4* | DBF | IRTKGGVEVHCCELPYHFLRFRSEHLYIFHGDLFPQG NKYHEVDVTMREITF |
| 2 | Arabidopsis | *RCY1* | RAD18 | IVFHRMECRRENLPLEHLDMYA |
|  |  |  | PMZ | RTTLCVRFRSEVVACGVVYAAARRFQVP |
| 3 | Barley | *Mla1* | BED | KMKPKCEWDILLQSLGSGLTEDNSLEEMRRILSFSYSNLPSHLKTCLLY |
| 4 | Barley | *Mla12* | BED | KDVSLRWDVFVLLYCGGARVGEAKEAEAAVRRALEAHPRHPRI |
| 5 | Barley | *Rpg1* | TTF | NGQYIAVKKLHLMPGLDDEEFKNEFNNLMRVRHQNIIPLVGYCHHTKQVLVEHNGKHVSARVEERYLCSEYLEGGSLDKHLSNE |
|  |  |  | C3H1 | WDRCYKIIKGICEGLHYLHNA |
| 6 | Flax | *M* | Rad18 | AKAVYNKISSHFDRCC |
| 7 | Potato | *Rx2* | BED | SKTLDEWQNVAENVRSVVSTDLEAKCMRVLALSYHHLPSHLKPCFLY |
| 8 | Potato | *Gpa2Rx1* | BED | GQRLDEWQRIGENVSSVVSTDPEAQCMRVLALSYHHLPSHLKPCFLY |
| 9 | Potato | *Gro 1.4* | CHCC | DVDPSTVRKQKSIFGEAFSKHEARFQEDKVQKWRAALEEAA |
|  |  |  | CDGSH | TLPKRIRLEKLEILVLTGCSKLRTFPEIEEK |
| 10 | Rice | *Pib* | U1 | LEKTSRIIVTTRKENIANHCSGKNGNVHNLKVLKH |
|  |  |  | TTF | DNSKVVKTWVKQVRDTAYDVEDSLQDFAVHLKRPSWWRFPRTLLERHRVAKQMKELRNKVEDVSQRNVRYHLIKGSAKATINS |
| 11 | Rice | *Pi-ta* | UBP | SCVVHHMVLNFIRCKSIEENFSITLDHSQTTVRHADKVRRLSL |
| 12 | Rice | *Pi36* | CHCC | KTPHSFVRFDLHGCESSSGASSFMPRLEHIEFSVDVRFLK |
| 13 | Rice | *Pi-k^h^* (*Pi54*) | NFX | CGCHELSCLPQ |
| 14 | Rice | *Piz-t* | PMZ | EISKLQCLHTLRCIGQFHYD |
| 15 | Rice | *pi21* | LIM | QCCRCDAKIRKVLGCLEEEYCIEKVEYDVKNNRVIVRGK |
| 16 | Rice | *Pi5-1* | TAZ | SLQYLNLSKCLKLEVLPQSFGQLAYLKSLNLSYCSDLKLLESFECLTSLRFLNLSNCSRLEYLPSCF |
| 17 | Rice | *Pi-2* | UBP | RDLSYNIEDCLDEFKVHVESQSLAKQLMKLGERHRIAVQIRNLKS |
| 18 | Sunflower | *pI8* | CHCC | PFHSGATGSRVIMTTRQQQLLKKMGFNHLDLLESLSHDDALSLLA |
|  |  |  | GATA | DKLKMVEVECYQGMEFPNWVGDPSFNRLVHVSLRACRKCTSLP |
|  |  |  | C2C2 | GSCKNMKAFADLQLPNLIRWRLWNCENLESF |
| 19 | Tobacco | *N* | C2H2 | LRCFVCTNYPWESFPSTFELKMLVHLQLRH |
| 20 | Tomato | *Mi-1* | UBR1 | ESWDYSTEQHWFPKLDCLTELETLCVGFKSSNTNHCGSSVATNRPWDFHFPSNLKE |
| 21 | Tomato | *Sw5-e* | UBP | PDECGTNLNLERLESHFLEFFQGNTASLSCNSESNDF |
| 22 | Tomato | *Cf-9* | PMZ | EICHLSKLHVLRICDQYGLS |
| 23 | Tomato | *I2C* | U1 | LQKLYISYCKKLVNGRKEWHLQRLTEL |
| 24 | Tomato | *Hero* | ZZ | DFYQLECNKTTKTEFLYTRYQVTVDRVTQFCFDLWTGKYRNYRY |
|  |  |  | UBR1 | DFCSVKAGKEKFFKLINSGDPFHASDFLHHRLTIHTDDKKCVLFNSNKCSAGS |
| 25 | Tomato | *Cf-4* | ZNF_C4 | NPNASDYCYDRRTLSWNKSTSCCSWDGVHCDETTGQVIE |
| 26 | Wheat | *Lr10* | C2C2 | KYCPPSKCSQHGPMIRYPLCLESSNTSSSSSCGCAGRS |
|  |  |  | ZZ | VSWDEGDCRECELSGRRCAFSSQRDREFCMPDPHGSHIK |
