# Supplementary material for: Comparative Analysis of Zinc Finger Proteins Involved in Plant Disease Resistance
Source: PLoS One. 2012 Aug 15;7(8):e42578. doi: 10.1371/journal.pone.0042578 (PMC3419713; doi:10.1371/journal.pone.0042578)
Supplement: Table S4 — Biophysical parameters of identified Zinc finger domains across various cloned R genes. (DOCX) [file pone.0042578.s005.docx]

| **Table S4** Biophysical parameters of identified Zinc finger domains across various cloned R-genes | | | | | | | |
| --- | --- | --- | --- | --- | --- | --- | --- |
|  |  |  |  |  |  |  |  |
| **S. No.** | **Disease resistance gene** | **Zinc Finger Type** | **Mol. Wt.** | **Thr. PI** | **Instability index** | **Aliphatic index** | **GRAVY**^*^ |
| 1 | *SSI4* | DBF | 6267.1 | 6.55 | 39.85 | 74.81 | -0.356 |
| 2 | *RCY1* | RAD18 | 2773.2 | 6.03 | 78.55 | 88.64 | -0.423 |
|  |  | PMZ | 3155.7 | 10.66 | 75.62 | 90.36 | 0.421 |
| 3 | *Mla1* | BED | 5689.6 | 6.76 | 70.05 | 95.51 | -0.31 |
| 4 | *Mla12* | BED | 4843.5 | 9.19 | 35.75 | 95.35 | -0.256 |
| 5 | *Rpg1* | TTF | 9771 | 6.4 | 27.19 | 86.9 | -0.674 |
|  |  | C3H1 | 2532.9 | 8.04 | 21.96 | 97.62 | -0.267 |
| 6 | *M* | Rad18 | 1842.1 | 8.87 | 22.71 | 55 | -0.331 |
| 7 | *Rx2* | BED | 5412.1 | 6.28 | 22.31 | 87.02 | -0.317 |
| 8 | *Gpa2Rx1* | BED | 5430.2 | 6.78 | 27.08 | 95.32 | -0.164 |
| 9 | *Gro 1.4* | CHCC | 4734.2 | 6.78 | 27.93 | 54.88 | -0.99 |
|  |  | CDGSH | 3654.4 | 9.1 | 83.53 | 122.58 | -0.235 |
| 10 | *Pib* | U1 | 3953.5 | 10.19 | 13.26 | 94.57 | -0.723 |
|  |  | TTF | 9905.3 | 10.22 | 42.73 | 78.55 | -0.896 |
| 11 | *Pi-ta* | UBP | 5021.8 | 8.89 | 42.76 | 99.53 | -0.121 |
| 12 | *Pi36* | CHCC | 4582.1 | 7.02 | 50.69 | 63.25 | -0.25 |
| 13 | *Pi-k^h^* (*Pi54*) | NFX | 1189.3 | 5.24 | 120.89 | 70.91 | 0.191 |
| 14 | *Piz-t* | PMZ | 2404.7 | 6.99 | 72.94 | 97.5 | -0.19 |
| 15 | *pi21* | LIM | 4604.4 | 8.37 | 37.94 | 89.74 | -0.5 |
| 16 | *Pi5-1* | TAJ | 7703 | 7.55 | 50.68 | 116.42 | 0.254 |
| 17 | *Pi-2* | UBP | 5312.1 | 8.15 | 57.8 | 101.78 | -0.604 |
| 18 | *PI8* | CHCC | 4978.7 | 7.42 | 36.4 | 99.78 | -0.124 |
|  |  | GATA | 4984.8 | 7.94 | 51 | 72.33 | -0.288 |
|  |  | C2C2 | 3698.2 | 8.05 | 69.56 | 81.94 | -0.342 |
| 19 | *N* | C2H2 | 3696.3 | 8.06 | 80.21 | 84.33 | 0.033 |
| 20 | *Mi-1* | UBR1 | 6521.1 | 5.13 | 27.96 | 46.96 | -0.752 |
| 21 | *Sw5-e* | UBP | 4166.4 | 4 | 28.21 | 55.41 | -0.649 |
| 22 | *Cf-9* | PMZ | 2327.7 | 6.99 | 42.43 | 131.5 | 0.24 |
| 23 | *I2C* | U1 | 3361 | 9.75 | 32.26 | 111.85 | -0.619 |
| 24 | *Hero* | ZZ | 5605.3 | 8.68 | 11.76 | 46.36 | -0.875 |
|  |  | UBR1 | 5942.7 | 8.58 | 6.37 | 60.75 | -0.43 |
| 25 | *Cf-4* | ZNF_C4 | 4442.7 | 4.68 | 57.52 | 37.44 | -0.928 |
| 26 | *Lr10* | C2C2 | 4010.5 | 8.76 | 79.95 | 33.42 | -0.555 |
|  |  | ZZ | 4527.9 | 5.48 | 67.76 | 30 | -1.072 |

*Grand Average of Hydropathicity
